# Nirmatrelvir/ritonavir or Molnupiravir for treatment of non-hospitalized patients with COVID-19 at risk of disease progression

**DOI:** 10.1371/journal.pone.0298254

**Published:** 2024-06-06

**Authors:** Adeel Ajwad Butt, Peng Yan, Obaid S. Shaikh

**Affiliations:** 1 VA Pittsburgh Healthcare System, Pittsburgh, Pennsylvania, United States of America; 2 Weill Cornell Medicine, New York, New York, United States of America; 3 Weill Cornell Medicine Qatar, Doha, Qatar; 4 Hamad Medical Corporation, Doha, Qatar; 5 University of Pittsburgh School of Medicine, Pittsburgh, Pennsylvania, United States of America; Tokai University School of Medicine, JAPAN

## Abstract

**Background:**

In randomized controlled trials, Nirmatrelvir/ritonavir (NMV/r) and Molnupiravir (MPV) reduced the risk of severe/fatal COVID-19 disease. Real-world data are limited, particularly studies directly comparing the two agents.

**Methods:**

Using the VA National COVID-19 database, we identified previously uninfected, non-hospitalized individuals with COVID-19 with ≥1 risk factor for disease progression who were prescribed either NMV/r or MPV within 3 days of a positive test. We used inverse probability of treatment weights (IPTW) to account for providers’ preferences for a specific treatment. Absolute risk difference (ARD) with 95% confidence intervals were determined for those treated with NMV/r vs. MPV. The primary outcome was hospitalization or death within 30 days of treatment prescription using the IPTW approach. Analyses were repeated using propensity-score matched groups.

**Results:**

Between January 1 and November 30, 2022, 9,180 individuals were eligible for inclusion (6,592 prescribed NMV/r; 2,454 prescribed MPV). The ARD for hospitalization/death for NMV/r vs MPV was -0.25 (95% CI -0.79 to 0.28). There was no statistically significant difference in ARD among strata by age, race, comorbidities, or symptoms at baseline. Kaplan-Meier curves did not demonstrate a difference between the two groups (p-value = 0.6). Analysis of the propensity-score matched cohort yielded similar results (ARD for NMV/r vs. MPV -0.9, 95% CI -2.02 to 0.23). Additional analyses showed no difference for development of severe/critical/fatal disease by treatment group.

**Conclusion:**

We found no significant difference in short term risk of hospitalization or death among at-risk individuals with COVID-19 treated with either NMV/r or MPV.

## Introduction

As of June 20, 2023, there were over 690 million reported cases of COVID-19 infection and over 6.9 million resulting deaths globally [1]. While COVID-19 is primarily a respiratory illness, it may also affect the cardiovascular, renal, gastrointestinal, hepatic, endocrine and neurologic systems [2–8]. Recovered individuals experience a higher incidence of acute myocardial infarction, [2, 9]; stroke, [9, 10] decline in renal function, [4] and diabetes [11].

In December 2021, two novel oral antiviral agents, Nirmatrelvir/ritonavir (NMV/r) and Molnupiravir (MPV), were granted Emergency Use Authorization (EUA) by the Food and Drug Administration (FDA) for treatment of early symptomatic patients with mild to moderate COVID-19 at high risk of progression to severe disease [12–15]. NMV/r is a SARS-CoV-2-3CL protease inhibitor, the enzyme that the coronavirus needs to replicate. It inhibits viral replication intracellularly at the proteolysis stage before viral RNA replication. NMV has to be administered with low-dose ritonavir, a potent CYP3A inhibitor with no activity against SARS-CoV-2 on its own, which slows the metabolism of NMV and prolongs its half-life. In randomized controlled trials and real-world studies, NMV/r has been associated with reduced mortality, hospitalization, and hospital length of stay [16–20]. While MPV treatment was also associated a significant reduction in hospitalization or death by day 29 compared with the placebo group In randomized controlled trials, [21] real-world studies of MPV have shown mixed results with some studies reporting a reduction in mortality and hospitalizations and other showing no benefit [22–24]. Observational studies including both treatments have shown both to be beneficial compared with untreated controls, though these studies have generally not compared the two agents against each other [25–27]. An observational study compared NMV/r and MPV against untreated controls in hospitalized patients and found a survival benefit associated with both drugs, but no reduction in intensive care unit (ICU) admission or the need for ventilatory support [28]. Of note, the European Medicines Agency did not recommend approval of MPV noting absence of compelling evidence of benefit of MPV in patients with COVID-19, leading the manufacture to withdraw its application for marketing authorization in Europe in June 2023 [29]. A randomized controlled trial directly comparing NMV/r to MPV is very unlikely due to logistic, financial, and ethical constraints. In the absence of such trials, rigorous observational studies can provide real-world evidence of their comparative effectiveness in patients with COVID-19. We undertook this study to determine the comparative effectiveness of NMV/r vs. MPV treatment upon the risk of hospitalization or death in a previously uninfected, non-hospitalized population at risk for disease progression.

## Methods

### Study setting

The Veterans Health Administration of the Department of Veterans Affairs (VA) created a national COVID-19 Shared Data Resource, which contains detailed demographic, clinical, laboratory, vital status, and episodes-of-care information on all Veterans with a laboratory-confirmed diagnosis of COVID-19 infection and recipients of a COVID-19 vaccine within the VA. Veterans who are tested or vaccinated outside VA are captured by patient self-report (presentation of a vaccination card) or through insurance claims data. The VA COVID-19 Shared Data Resource is updated regularly in real time with information derived from multiple validated sources [30–34].

### Study population

We used a matched cohort design for the current study using two approaches described below. Eligible individuals were those in the VA COVID-19 Shared Data Resource with at least two episodes of care in the VA healthcare system within the last 2 years, who had a first confirmed SARS-CoV-2 infection between January 1 and November 30, 2022, had at least one risk factor for progression to severe disease, and received either NMV/r or MPV within 3 days of their COVID-19 diagnosis. Those who were hospitalized or died before or within 24 hours of receiving NMV/r or MPV, those who received both NMV/r and MPV, and those who received monoclonal antibody for COVID-19 or remdesivir were excluded, as were those who received treatment≥ 3 days after the index diagnosis. We used an inverse probability of treatment weights (IPTW) based approach for our primary analysis, as detailed in our previous publications [35, 36]. Briefly, we fitted a logistic regression model for NMV/r or MPV prescription using age (5 year blocks), race, sex, body mass index, VA facility where diagnosis was made, vaccination status, and presence of diabetes, hypertension, cardiovascular disease, chronic kidney disease, chronic lung disease, and cancer diagnoses. The estimated probabilities from this model were used to compute inverse probability of treatment weights, which were used to weigh in subsequent analyses. To account for potential replications caused by IPTW, we used a robust (sandwich) variance estimator in Cox regression model, which yielded conservative 95% confidence intervals. Adequacy of weighting was tested by calculating the standardized mean difference for each variable after applying the weights. A value of <0.2 indicates good matching for the variable tested.

We conducted additional analyses to determine the validity of our primary results. Among the eligible population, we used propensity-score matching to identify those prescribed NMV/r and 1:1 matched controls prescribed MPV. Propensity-score matching was done on age, race, sex, body mass index, multiple comorbidities, site of diagnosis and vaccination status. We used matching without replacement using a caliper of 0.2SD. We calculated the ARD and 95% confidence intervals for hospitalization or death within 30 days overall, and for various subgroups.

Vaccination status was categorized based on the status at the time of COVID-19 diagnosis into individuals who were unvaccinated or who did not complete a primary series, those who completed a primary series, and those who completed a primary series and received at least one booster dose after that. Body mass index was calculated using the average of two most recent height and weight values. Comorbidities were retrieved from the VA National COVID-19 database, where they are identified based on International Classification of Diseases– 10^th^ edition (ICD-10) codes.

### Primary outcome measure

Our primary outcome measure was hospitalization or death within 30 days among those prescribed NMV/r vs. those prescribed MPV. Time-at-risk started from the date of treatment prescription in each group.

### Statistical analysis

We calculated the absolute risk difference (ARD) and associated 95% confidence intervals between the groups overall, and for sub-strata of the population by age, sex, body mass index, presence of various comorbidities, vaccination status, and presence of symptoms. Kaplan-Meier curves were generated to demonstrate the difference in outcomes over time among those treated with NMV/r or MPV. Logrank test was used to calculate p-values between groups. A p-value of <0.05 was considered statistically significant.

### Additional analyses

We repeated all analyses comparing NMV/r vs. MPV for development of severe, critical or fatal disease. Severe or critical disease were defined as need for intensive care unit admission or mechanical ventilation, or death. In addition, we determined the hazards ratios for the risk of developing the primary outcome using Cox proportional hazards analysis.

### Ethics statement

The study was granted an exempt status by the Institutional Review Board at the VA Pittsburgh Healthcare System (Study Number 1617395–6). Since there was no contact with any of the participants, and due to its exempt status, the informed consent requirement was not applicable.

## Results

**Fig 1:**
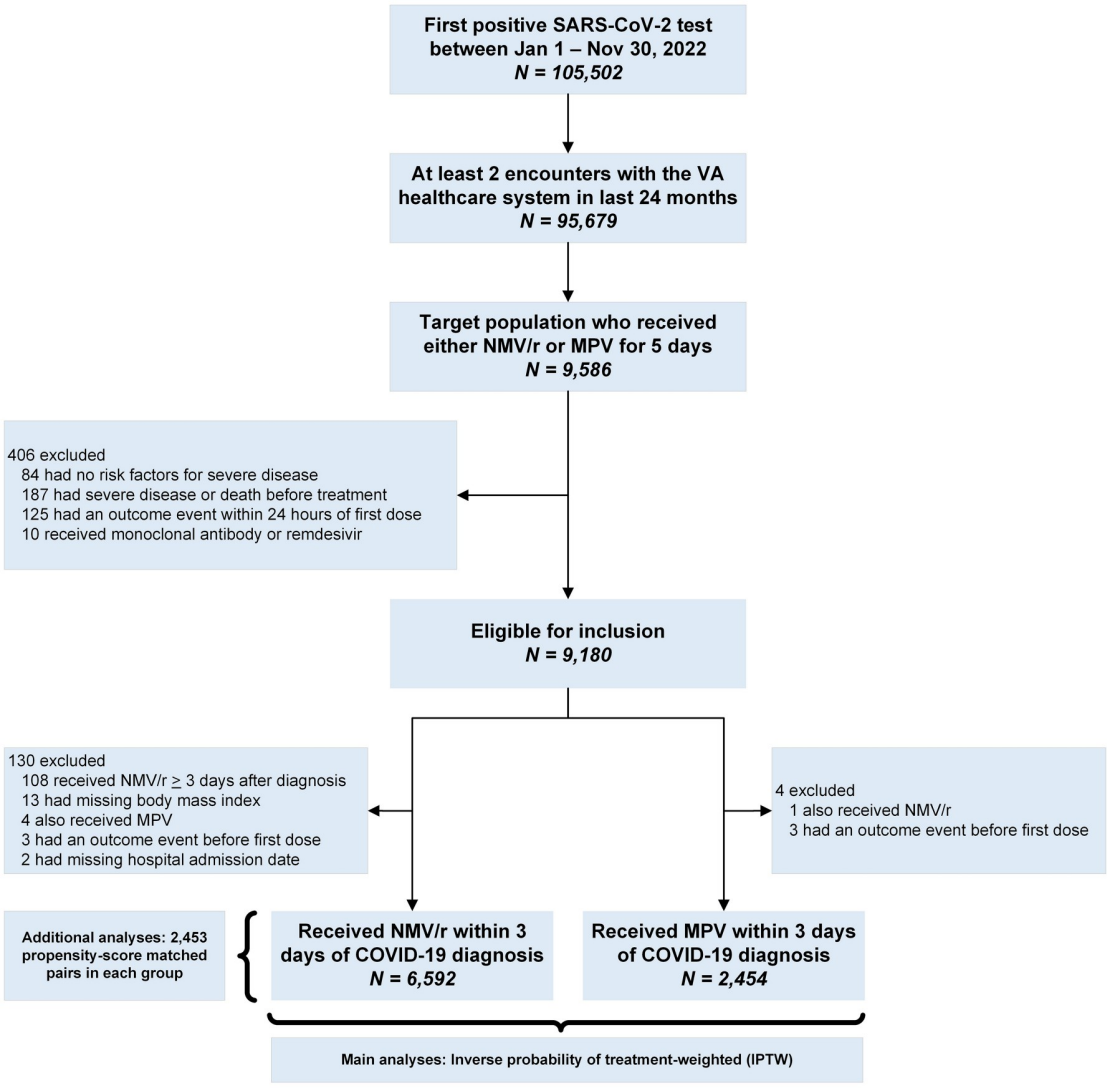
Cohort construction. NMV/R, nirmatrelvir/ritonavir; MPV, molnupiravir; Inverse probability of treatment weights. IPTW and matching done on age (5-year blocks); race; sex; BMI groups; comorbidities; VA station where treatment prescribed; vaccination status.

**Fig 2:**
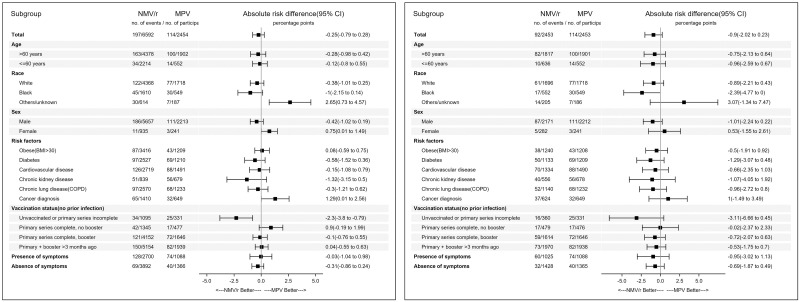
Incidence of hospitalization or death within 30 days and absolute risk difference among patients who received Nirmatrelvir/ritonavir or Molnupiravir. Panel A: Inverse probability of treatment weighted groups; Panel B: Propensity-score matched groups.

**Fig 3:**
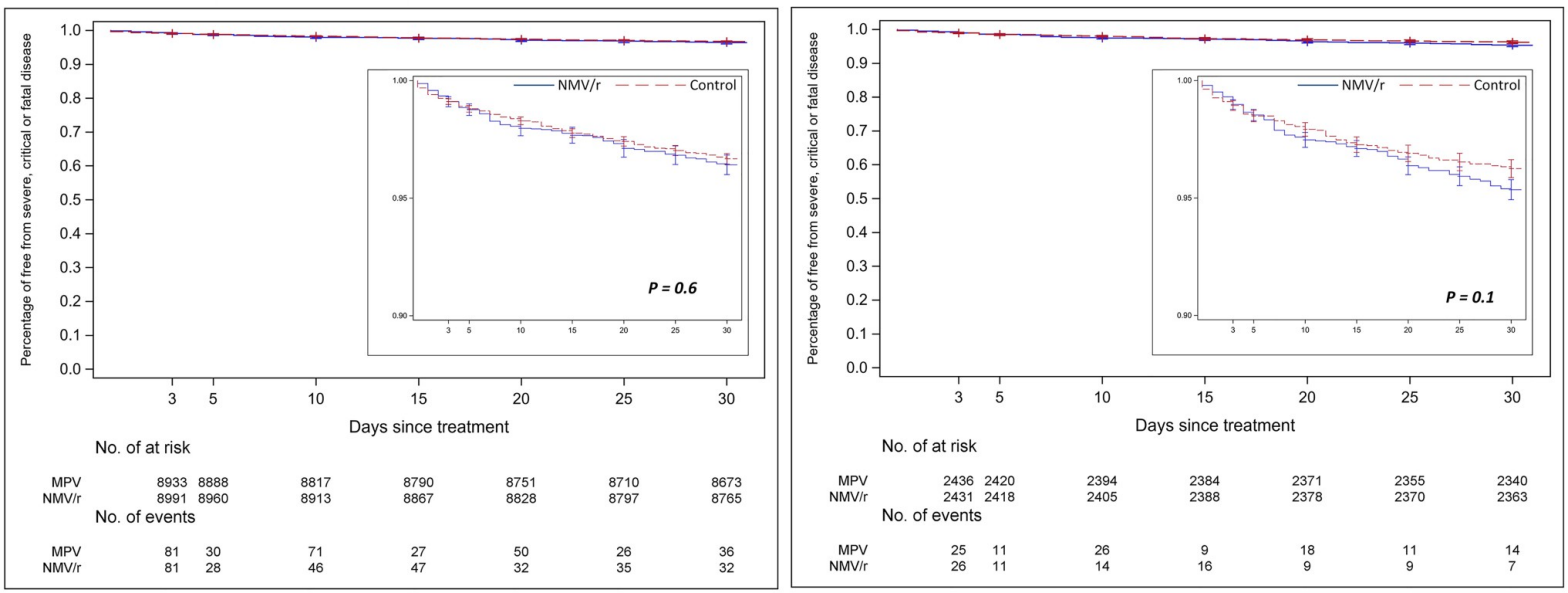
Kaplan-Meier curves depicting proportion of individuals without hospitalization or death among those treated with Nirmatlevir/ritonavir or Molnupiravir. Panel A: Inverse probability of treatment weighted groups; Panel B: Propensity-score matched groups.

Among 105,502 individuals who tested positive during the study period, 9,180 were eligible for inclusion in the final analyses. (Fig 1) Among those, the primary analyses were conducted 6,592 individuals prescribed NMV/r and 2,454 prescribed MPV using the inverse probability of treatment weights. (Fig 1) The standardized mean difference values before and after inverse probability of treatment weighting are provided in S1 Fig in S1 File. The median age in the IPTW groups was 67 years, 87% were male, 23% were Black. Median body mass index was 30 kg/m^2^, median Charlson comorbidity index was 2, and approximately 16% were unvaccinated against COVID-19. (Table 1) Median number of days from diagnosis to prescription, and from onset of symptoms to prescription among symptomatic individuals was 0 days (IQR 0,1). The absolute risk difference (ARD) for hospitalization or death within 30 days among patients who received NMV/r vs those who received MPV was -0.25 (95% CI -0.79 to 0.28). (Fig 2, Panel A) There was no statistically significant difference in ARD among strata by age, race, comorbidities, or symptoms at baseline. (Fig 2, Panel A) Absolute risk difference for hospitalization or death among NMV/r treated vs. MPV treated was -2.3 (95% CI -3.8 to -0.79) for those who were unvaccinated or did not complete a primary series, and 0.9 (95% CI -0.19 to 1.99) for those who had completed a primary series but not received a booster dose. There was no significant difference among those who had received a booster dose after completing a primary series. Kaplan-Meier curves depicting the proportion of individuals without hospitalization or death among those treated with NMV/r or MPV is shown in Fig 3, Panel A and did not demonstrate a difference between the two groups (logrank p-value = 0.6).

**Table 1:** Baseline characteristics of the Nirmatrelvir/ritonavir (NMV/r) and Molnupiravir (MPV) analysis cohort.

	Before IPTW			After IPTW		
	NMV/r group	MPV group		NMV/r group	MPV group	
	*N = 6592*	*N = 2454*	SMD*	*N = 6592*	*N = 2454*	SMD*
Median age, years, (IQR)	66 (56,74.4)	70.2 (61.1,75.7)	0.29	67.2 (57.4,74.8)	67 (57.9,74.8)	0
Male sex, %	85.82%	90.18%	0.13	87%	86.65%	-0.01
Race, %			0.09			0.02
White	66.26%	70.01%		67.39%	68.07%	
Black	24.42%	22.37%		23.75%	22.91%	
Other/unknown	9.31%	7.62%		8.86%	9.02%	
Median body mass index, kg/m^2^, (IQR)	30.2 (26.6,34.4)	29.9 (26.4,34.2)	-0.04	30.2 (26.5,34.3)	30 (26.4,34.4)	0
Median Charlson Comorbidity Index score, (IQR)	2 (0,3)	3 (1,5)	0.43	2 (1,3)	2 (1,4)	0.05
Comorbidities, %						
Obesity (BMI >30 kg/m^2^)	51.82%	49.27%	-0.05	51.43%	49.96%	-0.03
Diabetes	38.33%	49.31%	0.22	41.37%	41.76%	0.01
Hypertension	68.86%	81.95%	0.31	72.44%	72.34%	0
Cardiovascular disease	41.25%	60.76%	0.4	46.62%	46.88%	0.01
Chronic kidney disease	12.73%	27.67%	0.38	16.92%	17.05%	0
Chronic lung disease	38.99%	50.24%	0.23	42.18%	42.19%	0
Cancer diagnosis	21.39%	26.45%	0.12	22.87%	23.24%	0.01
Vaccination status at baseline			0.12			0
Unvaccinated or primary series incomplete	16.61%	13.49%		15.77%	16.1%	
Primary series complete	20.4%	19.44%		20.17%	20.49%	
Primary series + booster	62.99%	67.07%		64.05%	63.41%	
Median days (IQR) from symptoms to prescription	0 (0,1)	0 (0,1)	-0.04	0 (0,1)	0 (0,1)	-0.03
Median days (IQR) from diagnosis to prescription	0 (0,1)	0 (0,1)	-0.02	0 (0,1)	0 (0,1)	-0.01

NMV/r, Nirmatrelvir/ritonavir; MPV, Molnupiravir; IPTW, inverse probability of treatment weights; SMD, standardized mean difference; BMI, body mass index; IQR, inter quartile range.

### Additional analyses

The main analyses were repeated on a propensity-score matched groups that included 2,453 matched pairs. The standardized mean difference values before and after propensity-score matching are shown in S2 Fig in S1 File indicating good matching on the variables tested. The baseline characteristics of the study population before and after propensity-score matching are shown in S1 Table in S1 File. There was no difference in the primary outcome among the two groups (ARD -0.9, 95% CI -2.02 to 0.23). (Fig 2, Panel B) Subgroup analyses by age, race, sex, comorbidities, vaccination status, or presence or symptoms also did not demonstrate any difference among those treated with NMV/r or MPV. Kaplan-Meier curves depicting the proportion of individuals without hospitalization or death among those treated with NMV/r or MPV also did not demonstrate a difference between the two groups (logrank p-value = 0.1). (Fig 3, Panel B).

We repeated all analyses with severe, critical, or fatal disease within 30 days of treatment initiation as the primary outcome. These results mirrored the corresponding primary analyses and are presented in S3 and S4 Figs in S1 File. We also determined the hazards of developing the primary outcome of interest using the Cox proportional hazards analysis, which also confirmed the results of the primary analysis. (S5 Fig, panels A and B in S1 File).

### Data access

The data for this study were accessed over an extended period during 2022 and 2023. The study was considered exempt from review by the Institutional Review Board at VA Pittsburgh Healthcare System. The authors did not have access to information that could directly identify participants included in the analyses during or after data collection.

## Discussion

Data comparing NMV/r vs. MPV are scant. Our comparison of the two antivirals against COVID-19 demonstrate that they have comparable effect in reducing the risk of hospitalization or death in non-hospitalized individuals with at least one risk for progression of disease. Recently, a small observational study noted that both drugs demonstrated effectiveness against hospitalization or death, and time to first negative COVID-19 test [37].

Pivotal randomized clinical trials of NMV/r and MPV demonstrated efficacy of both antivirals compared with placebo in reducing risk of hospitalization or death among non-hospitalized individuals at risk of disease progression when administered early in the course of COVID-19 [16, 21]. However, subsequent observational studies have shown mixed results, particularly for MPV. The PANORAMIC trial was a large, multicenter, open labeled, platform adaptive randomized controlled trial, which failed to show any benefit of MPV in reducing hospitalization or death among high-risk individuals [24]. In another study emulating a target trial comparing either NMV/r or MPV versus non-initiation of these treatments, both agents reduced all-cause mortality among hospitalized patients. However, there was no reduction in the need for intensive care unit admission or mechanical ventilation [28]. The use of NMV/r has been associated with more consistent results in improving clinical outcomes [20]. To our knowledge, no published studies have directly compared these two antivirals in the same eligible population. Since a gold-standard randomized, controlled trial comparing these two agents is extremely unlikely due to logistic and financial constraints, a rigorously conducted observational study may provide clinically meaningful information. We used several analytical approaches to match the groups receiving NMV/r or MPV to reduce selection bias and assignment of one treatment over the other. All analyses demonstrated no significant difference in the risk of hospitalization or death among non-hospitalized individuals with COVID-19 who were treated with NMV/r or MPV. Our study population included non-hospitalized patients with at least one risk factor for progression to severe disease. Furthermore, no difference in the two antivirals were observed for the development of severe, critical or fatal disease.

Some important differences between NMV/r and MPV should be considered when prescribing these agents. NMV/r is not indicated in individuals with severe renal impairment (eGFR < 30 mL/min), while dose reduction is recommended in those with eGFR between 30–60 mL/min. No dose adjustment is recommended in individuals with mild to moderate hepatic impairment (Child-Pugh Class A or B). Since Nirmatrelvir must be co-administered with ritonavir, extreme caution must be observed in individuals taking other drugs metabolized by CYP3A. No dose adjustments or drug interactions are listed for MPV in the prescribing information (package insert) based on the limited data available.

Several limitations should be considered when interpreting these results. Since the treatment assignment was not randomized, there is a risk of selection bias and residual confounding. Treatment assignment was dependent upon the choice of individual prescribers. Information on SARS-CoV-2 variants was not available. There is a possibility of previously undiagnosed infection among the study population, which may have conferred varying level of immunity. There is a small possibility on incomplete capture of hospitalizations if care was provided outside the VA healthcare system. Some comorbidities like chronic kidney disease, chronic lung disease, and diabetes have a wide spectrum of severity which may affect outcomes differently. For example, an individual with stable stage 3 chronic kidney disease may be affected quite differently than an individual with stage 5 disease who is on chronic hemodialysis. Such variations in disease severity were not considered in our study due to lack of sufficient data for accurate disease severity classification. Finally, we did not determine the effectiveness of either antiviral vs. no treatment.

We employed several strategies to mitigate the limitations noted above. To minimize bias due to non-random selection of the antiviral agent, we used inverse probability of treatment weights. We also used a propensity-score matched approach to balance the two groups based on their baseline characteristics. Both approaches yielded study groups that were well matched on multiple demographic and clinical characteristics. We included those individuals in our study who had at least one VA encounter within the previous two years to minimize persons who receive care outside the VA healthcare system. It should be noted that our study population was predominantly male, which is reflective of the population served by the VA healthcare system.

In summary, we found no significant difference in short term risk of hospitalization or death, or severe, critical, or fatal disease in non-hospitalized individuals with COVID-19 at risk of disease progression who were treated with either NMV/r or MPV.

## Supporting information

S1 File This article includes supplementary/supporting data titled “supplementary analyses”. (DOCX)
