# Supplementary material for: Nirmatrelvir/ritonavir or Molnupiravir for treatment of non-hospitalized patients with COVID-19 at risk of disease progression
Source: PLoS One. 2024 Jun 6;19(6):e0298254. doi: 10.1371/journal.pone.0298254 (PMC11156403; doi:10.1371/journal.pone.0298254)
Supplement: S1 File — (DOCX) [file pone.0298254.s001.docx]

**Supplementary Analyses**

**Article Title:** Nirmatrelvir/ritonavir or Molnupiravir for Treatment of Comparing Outcomes in Non-hospitalized Patients with COVID-19 Treated with Nirmatrelvir/ritonavir or Molnupiravir

**Authors:** Butt AA, Yan P, Shaikh OS

**Contents:**

Supplementary table 1. Baseline characteristics before and after propensity-score matching.

Supplementary figure 1. Standardized mean difference values before and after inverse probability of treatment weighting. A value of <0.2 indicates good balancing between groups.

Supplementary figure 2. Standardized mean difference values before and after propensity-score matching. A value of <0.2 indicates good balancing between groups.

Supplementary figure 3. Incidence of severe, critical, or fatal disease within 30 days and absolute risk difference among patients who received Nirmatrelvir/ritonavir or Molnupiravir. Panel A: Inverse probability of treatment weighted groups; Panel B: Propensity-score matched groups.

Supplementary figure 4. Kaplan-Meier curves depicting proportion of individuals without severe, critical, or fatal disease among those treated with Nirmatlevir/ritonavir or Molnupiravir. Panel A: Inverse probability of treatment weighted groups; Panel B: Propensity-score matched groups

Supplementary figure 5. Adjusted hazards ratios for development of hospitalization or death within 30 days among patients who received Nirmatrelvir/ritonavir or Molnupiravir. Panel A: Inverse probability of treatment weighted groups; Panel B: Propensity-score matched groups.

Supplementary table 1. Baseline characteristics before and after propensity-score matching.

|  | Before Match | | | | PS match | | | |
| --- | --- | --- | --- | --- | --- | --- | --- | --- |
|  | NMV/r group | MPV group |  | NMV/r group | | MPV group |  |  |
|  | *N=6592* | *N=2454* | SMD* | *N=2453* | | *N=2453* | SMD* |  |
| Median age, years, (IQR) | 66 (56,74.4) | 70.2 (61.1,75.7) | 0.29 | 69.1 (59.7,75.5) | | 70.2 (61.1,75.7) | 0.09 |  |
| Male sex, % | 85.82% | 90.18% | 0.13 | 88.5% | | 90.18% | 0.05 |  |
| Race, % |  |  | 0.09 |  | |  | 0.02 |  |
| White | 66.26% | 70.01% |  | 69.14% | | 70.04% |  |  |
| Black | 24.42% | 22.37% |  | 22.5% | | 22.38% |  |  |
| Other/unknown | 9.31% | 7.62% |  | 8.36% | | 7.58% |  |  |
| Median body mass index, kg/m^2^, (IQR) | 30.2 (26.6,34.4) | 29.9 (26.4,34.2) | -0.04 | 30.1 (26.4,34.3) | | 29.9 (26.4,34.2) | -0.01 |  |
| Median Charlson Comorbidity Index score, (IQR) | 2 (0,3) | 3 (1,5) | 0.43 | 2 (1,4) | | 3 (1,5) | 0.17 |  |
| Comorbidities, % |  |  |  |  | |  |  |  |
| Obesity (BMI >30 kg/m^2^) | 51.82% | 49.27% | -0.05 | 50.55% | | 49.25% | -0.03 |  |
| Diabetes | 38.33% | 49.31% | 0.22 | 46.19% | | 49.29% | 0.06 |  |
| Hypertension | 68.86% | 81.95% | 0.31 | 78.88% | | 81.94% | 0.08 |  |
| Cardiovascular disease | 41.25% | 60.76% | 0.4 | 54.38% | | 60.74% | 0.13 |  |
| Chronic kidney disease | 12.73% | 27.67% | 0.38 | 22.67% | | 27.64% | 0.11 |  |
| Chronic lung disease | 38.99% | 50.24% | 0.23 | 46.47% | | 50.22% | 0.08 |  |
| Cancer diagnosis | 21.39% | 26.45% | 0.12 | 25.44% | | 26.46% | 0.02 |  |
| Vaccination status at baseline |  |  | 0.12 |  | |  | 0.07 |  |
| Unvaccinated or primary series incomplete | 16.61% | 13.49% |  | 14.68% | | 13.49% |  |  |
| Primary series complete | 20.4% | 19.44% |  | 19.53% | | 19.4% |  |  |
| Primary series + booster | 62.99% | 67.07% |  | 65.8% | | 67.1% |  |  |
| Median days (IQR) from symptoms to prescription | 0 (0,1) | 0 (0,1) | -0.04 | 0 (0,1) | | 0 (0,1) | -0.04 |  |
| Median days (IQR) from diagnosis to prescription | 0 (0,1) | 0 (0,1) | -0.02 | 0 (0,1) | | 0 (0,1) | -0.04 |  |

NMV/r, Nirmatrelvir/ritonavir; MPV, Molnupiravir; IPTW, inverse probability of treatment weights; SMD, standardized mean difference; BMI, body mass index; IQR, inter quartile range.

Supplementary figure 1. Standardized mean difference values before and after inverse probability of treatment weighting. A value of <0.2 indicates good balancing between groups.


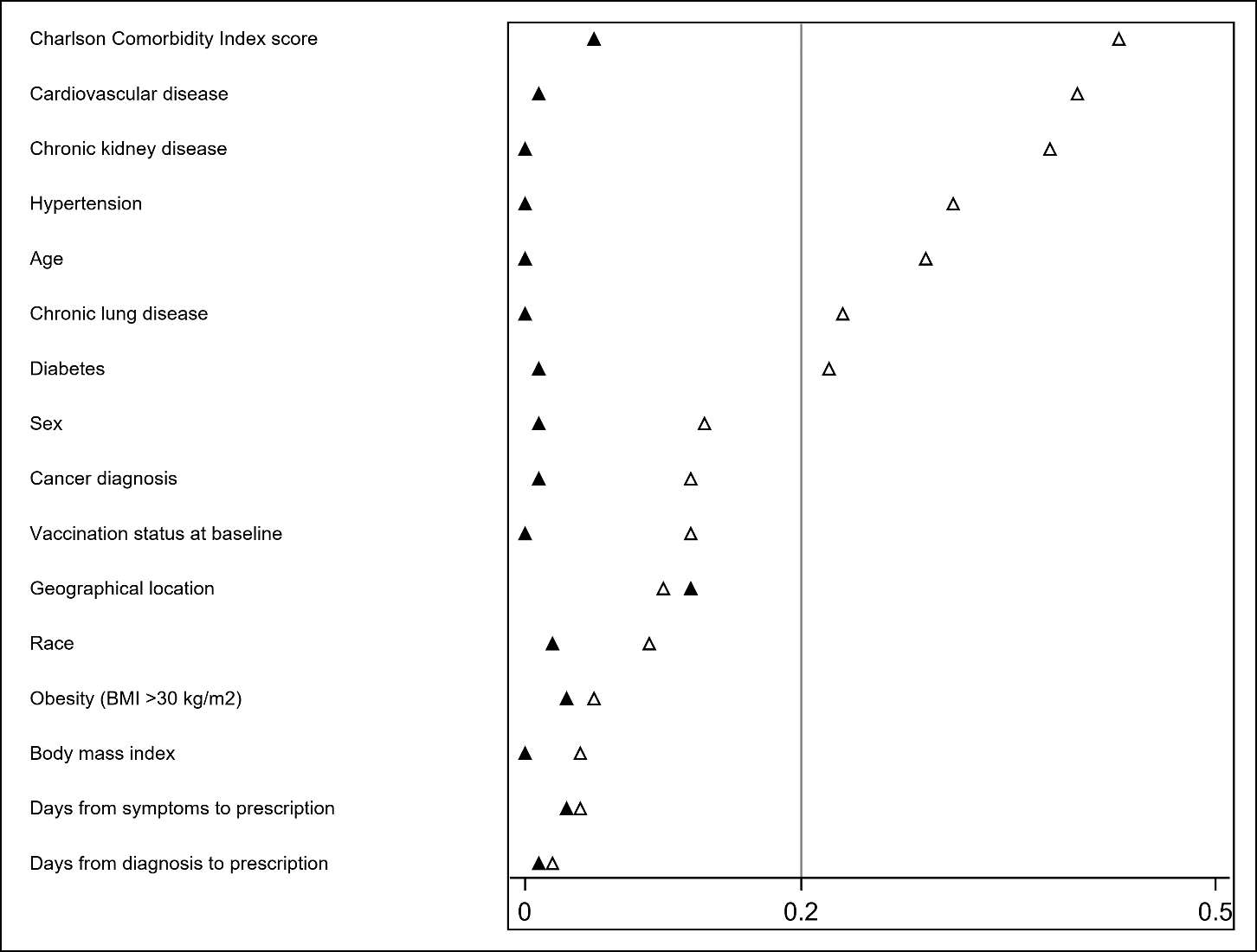


△ Before applying inverse probability of treatment weights, ▲ After applying inverse probability of treatment weights

Supplementary figure 2. Standardized mean difference values before and after propensity-score matching. A value of <0.2 indicates good balancing between groups.


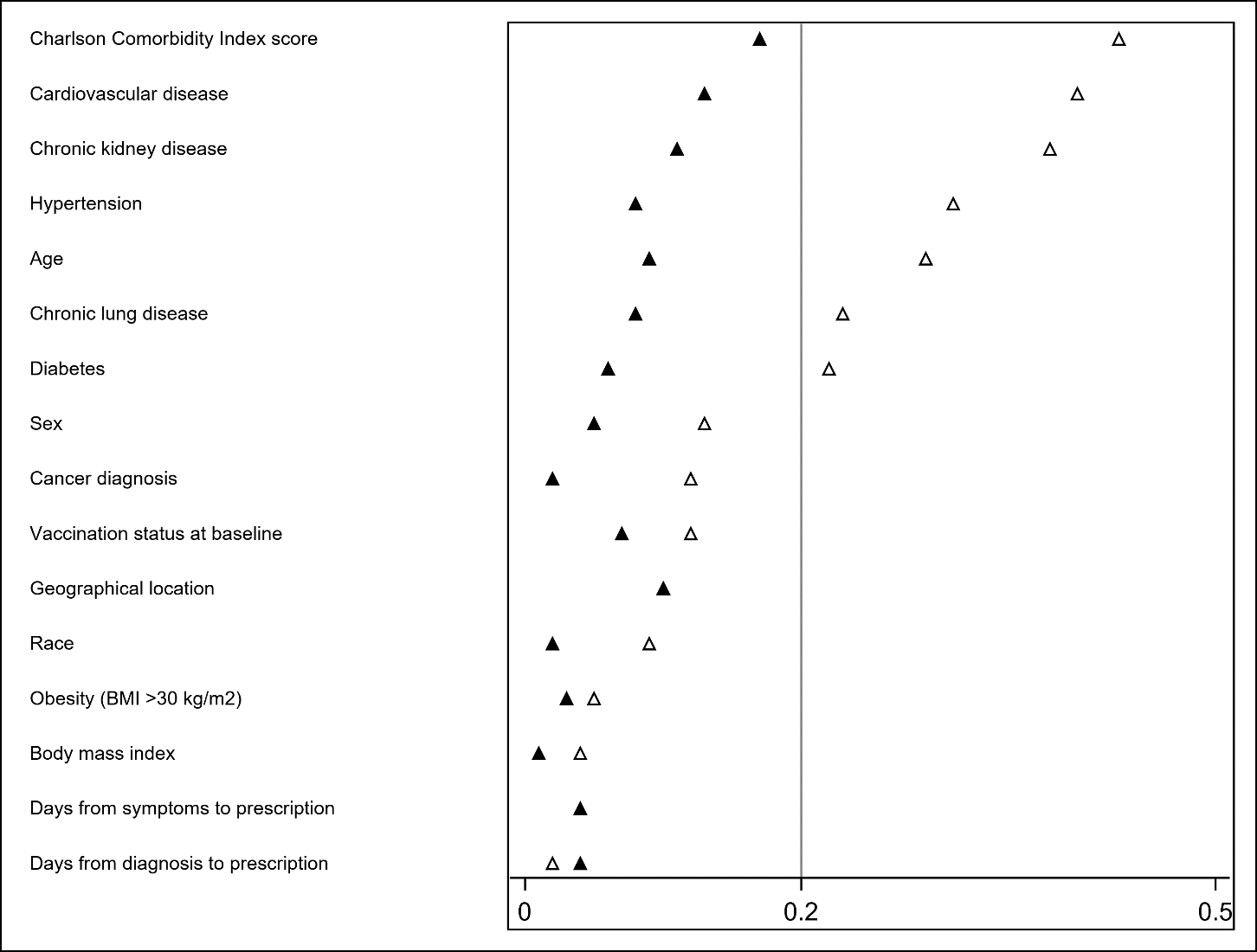


△ Before matching, ▲ After propensity-score matching

Supplementary figure 3. Incidence of severe, critical, or fatal disease within 30 days and absolute risk difference among patients who received Nirmatrelvir/ritonavir or Molnupiravir. Panel A: Inverse probability of treatment weighted groups; Panel B: Propensity-score matched groups.


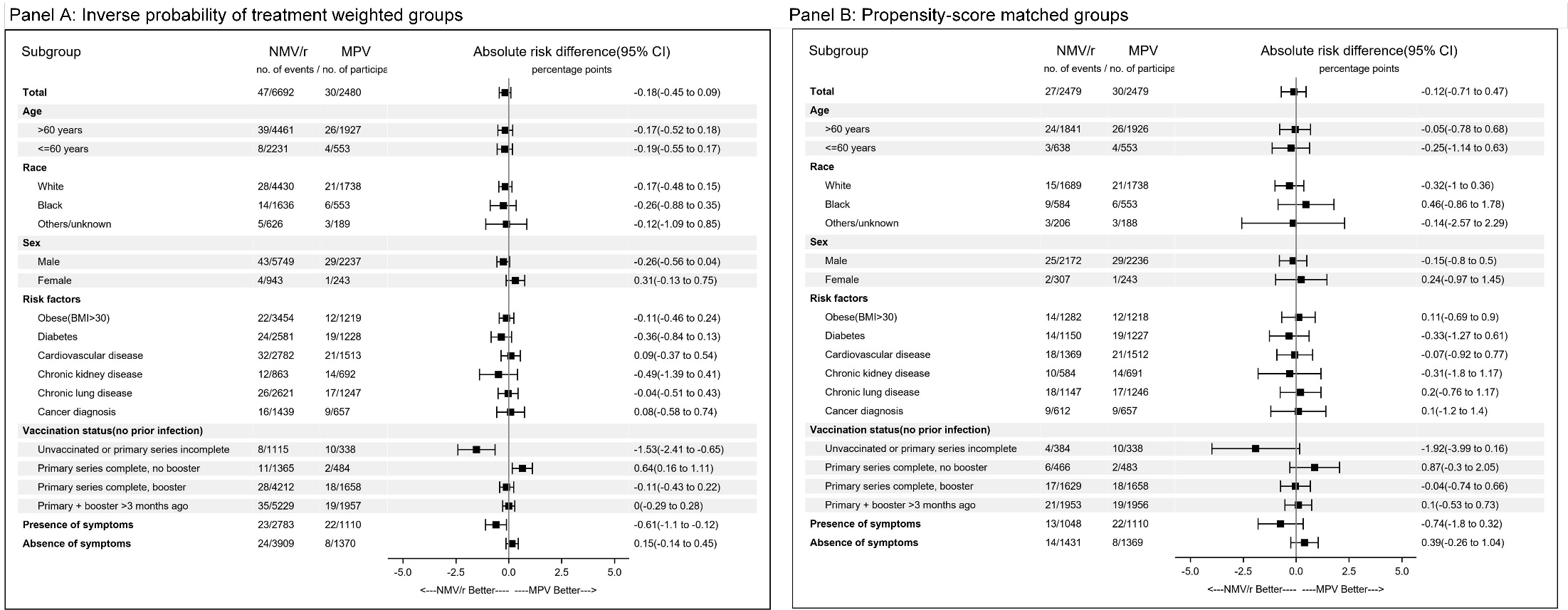


NMV/r, Nirmatrelvir/ritonavir; MPV, Molnupiravir; BMI, body mass index.

Supplementary figure 4. Kaplan-Meier curves depicting proportion of individuals without severe, critical, or fatal disease among those treated with Nirmatlevir/ritonavir or Molnupiravir. Panel A: Inverse probability of treatment weighted groups; Panel B: Propensity-score matched groups


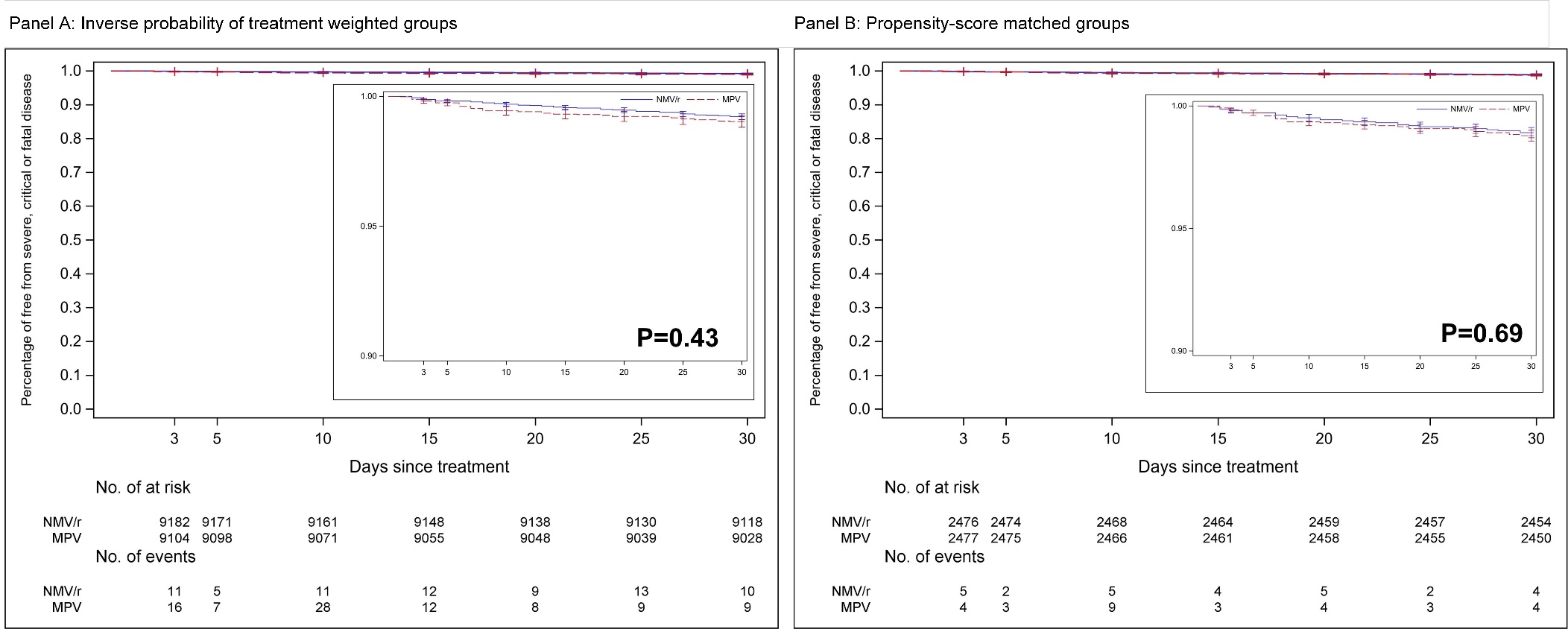


NMV/r, Nirmatrelvir/ritonavir; MPV, Molnupiravir.

Supplementary figure 5. Adjusted hazards ratios for development of hospitalization or death within 30 days among patients who received Nirmatrelvir/ritonavir or Molnupiravir. Panel A: Inverse probability of treatment weighted groups; Panel B: Propensity-score matched groups.


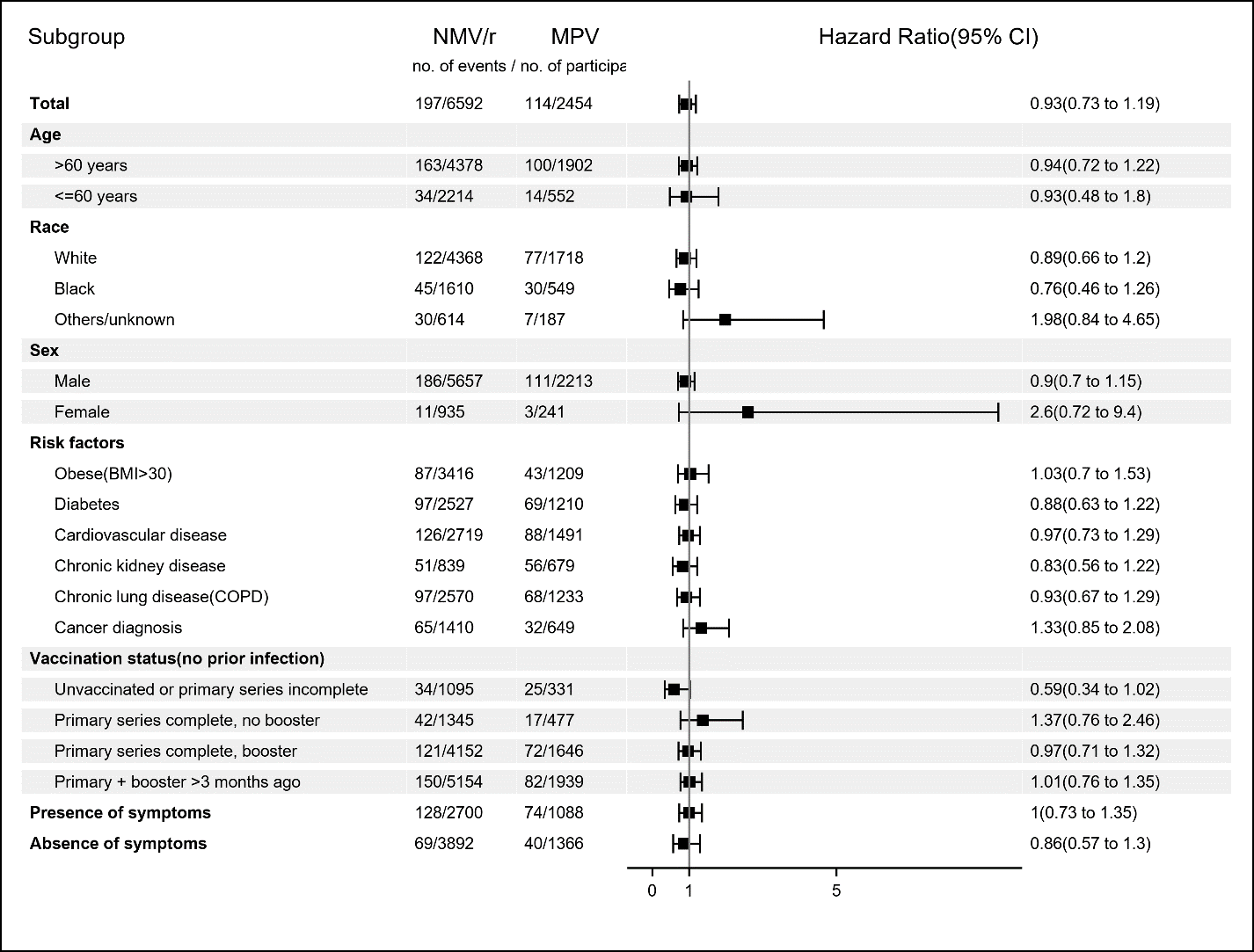

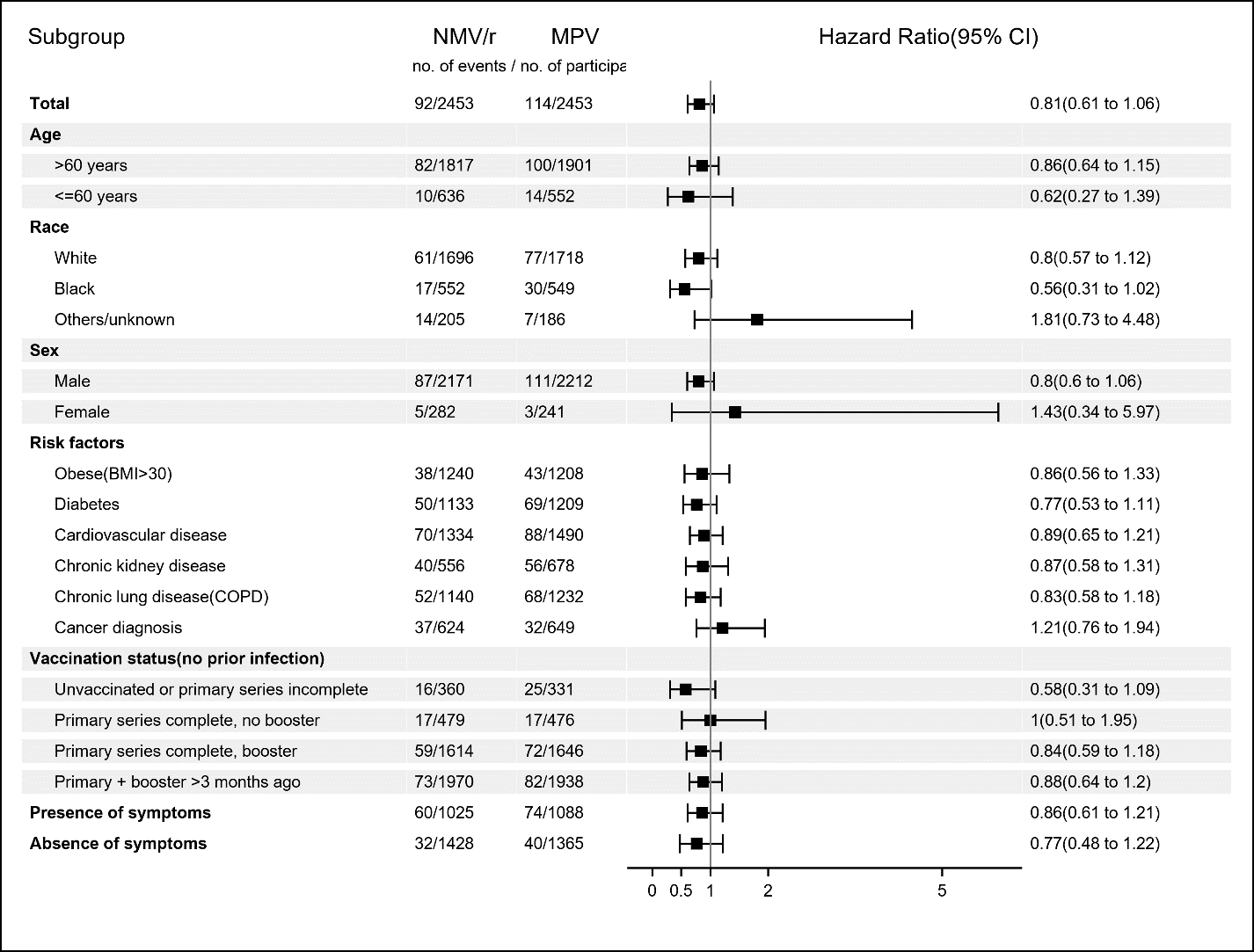


NMV/r, Nirmatrelvir/ritonavir; MPV, Molnupiravir; BMI, body mass index.
